# Structural studies on radiopharmaceutical DOTA-minigastrin analogue (CP04) complexes and their interaction with CCK2 receptor

**DOI:** 10.1186/s13550-018-0387-3

**Published:** 2018-04-16

**Authors:** Piotr F. J. Lipiński, Piotr Garnuszek, Michał Maurin, Raphael Stoll, Nils Metzler-Nolte, Artur Wodyński, Jan Cz. Dobrowolski, Marta K. Dudek, Monika Orzełowska, Renata Mikołajczak

**Affiliations:** 1 Neuropeptides Department, Mossakowski Medical Research Centre Polish Academy of Sciences, Pawińskiego 5 Str., 02-106 Warszawa, Poland; 2 Radioisotope Centre POLATOM, National Centre for Nuclear Research, A. Sołtana 7 Str, 05-400 Otwock, Poland; 3 Faculty of Chemistry and Biochemistry, Ruhr University of Bochum, Universitätsstr. 150, 44780 Bochum, Germany; 4 Świerk Computing Centre, National Centre for Nuclear Research, A. Sołtana 7 Str., 05-400 Otwock, Poland; 5 Institut für Chemie, Theoretische Chemie/Quantenchemie, Technische Universität Berlin, Sekr. C7, Strasse des 17. Juni 135, 10623 Berlin, Germany; 6 Institute of Nuclear Chemistry and Technology, Dorodna 16 Street, 03-195 Warszawa, Poland; 7 National Medicines Institute, Chełmska 30/34 Str., 00-725 Warszawa, Poland; 8 Centre of Molecular and Macromolecular Studies Polish Academy of Sciences, Sienkiewicza 112, 90-363 Lodz, Poland

**Keywords:** Minigastrin analogue, Medullary thyroid carcinoma, Cholecystokinin receptor subtype 2, Molecular docking

## Abstract

**Background:**

The cholecystokinin receptor subtype 2 (CCK-2R) is an important target for diagnostic imaging and targeted radionuclide therapy (TRNT) due to its overexpression in certain cancers (e.g., medullary thyroid carcinoma (MTC)), thus matching with a theranostic principle. Several peptide conjugates suitable for the TRNT of MTC have been synthesized, including a very promising minigastrin analogue DOTA-(DGlu)_6_-Ala-Tyr-Gly-Trp-Met-Asp-Phe-NH_2_ (CP04). In this contribution, we wanted to see whether CP04 binding affinity for CCK-2R is sensitive to the type of the complexed radiometal, as well as to get insights into the structure of CP04-CCK2R complex by molecular modeling.

**Results:**

In vitro studies demonstrated that there is no significant difference in CCK-2R binding affinity and specific cellular uptake between the CP04 conjugates complexed with [^68^Ga]Ga^3+^ or [^177^Lu]Lu^3+^. In order to investigate the background of this observation, we proposed a binding model of CP04 with CCK-2R based on homology modeling and molecular docking. In this model, the C-terminal part of the molecule enters the cavity formed between the receptor helices, while the N-terminus (including DOTA and the metal) is located at the binding site outlet, exposed in large extent to the solvent. The radiometals do not influence the conformation of the molecule except for the direct neighborhood of the chelating moiety.

**Conclusions:**

The model seems to be in agreement with much of structure-activity relationship (SAR) studies reported for cholecystokinin and for CCK-2R-targeting radiopharmaceuticals. It also explains relative insensitivity of CCK-2R affinity for the change of the metal. The proposed model partially fits the reported site-directed mutagenesis data.

## Background

With the enormous significance of cancer therapies for public health and the limited success of the currently available treatments (e.g., chemotherapy, external radiotherapy), there is an urgent need for development of novel—safer and more effective—strategies. One of the most intensively advancing and most promising directions in this realm is the *t*argeted *r*adio*n*uclide *t*herapy (TRNT). Its principle is to selectively accumulate the radionuclide-carrying pharmaceutical only in the tumor tissues so that the toxic effect of ionizing radiation may be restricted to the diseased cells with the negative impact on healthy ones being minimized. The same mechanism may be applied for the targeted diagnosis of cancers if the radionuclide used is a γ-emitter or positron emitter. In this case, a small dose of a radiopharmaceutical accumulated selectively in the cancer tissues precisely localizes the tumors. The combination of diagnostic and therapeutic properties renders radiopharmaceuticals ideal candidates for theranostics, i.e., allowing the early detection of the disease and patient stratification.

The radionuclide is (most often) brought to the site of action in the form of a complex with a chelator attached to a vector (small organic molecule, peptide, antibody, etc.) that is able to selectively target a specific type of cells. The selectivity here is possible thanks to the fact that many types of cancers overexpress particular proteins, especially receptors, on their surfaces. A properly chosen vector—with high affinity for an overexpressed receptor—enables concentration of the therapeutic radionuclide in the desired area and brings about the enhancement of its therapeutic action.

**Fig. 1:**
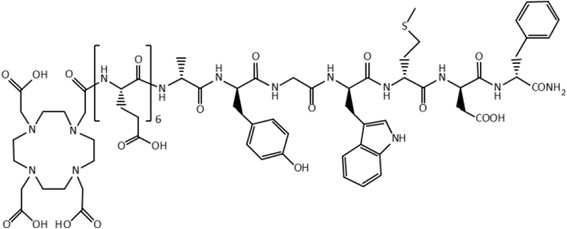
Structure of CP04

Among the cancer types which may benefit due to the treatment and diagnosis offered by targeted radionuclide delivery are medullary thyroid carcinomas (MTC). MTC belong still to one of the most challenging cancers. They are especially difficult to handle, especially in their advanced stages. Due to the high overexpression of the cholecystokinin receptor subtype 2 (CCK-2R) on MTC cells (incidence of > 90%) [1–3], it has been proposed that CCK-2R targeting peptides may serve as vectors for diagnostic imaging and TRNT of MTC. Several suitable peptide conjugates have been now reported with some of them showing high receptor affinity and ability for internalization [4]. One of these is a minigastrin (MG; H-Leu-(Glu)_5_-Ala-Tyr-Gly-Trp-Met-Asp-Phe-NH_2_) analogue CP04: DOTA-(DGlu)_6_-Ala-Tyr-Gly-Trp-Met-Asp-Phe-NH_2_ (Fig. 1). Its radiolabeled complexes have been studied with respect to stability [5], receptor binding, internalization [4], in vivo tumor targeting in animals [6], and kidney retention [7]. These encouraging results prompted further clinical evaluation of [^111^In]In-CP04 within the ERA-NET project GRAN-T-MTC [8, 9].

Due to the properties of the macrocyclic chelator DOTA (1,4,7,10-tetraazacyclododecane-1,4,7,10-tetraacetic acid) N-terminally attached to the peptide sequence, CP04 can be radiolabeled with [^111^In]In^3+^ or [^68^Ga]Ga^3+^ for imaging or with [^90^Y]Y^3+^ and [^177^Lu]Lu^3+^ for therapy of MTC. However, another aspect of CP04 radiopharmaceutical properties that has remained only partially examined so far is whether their binding affinity to CCK-2R is influenced by the type of the incorporated radiometal. Such sensitivity has been demonstrated for, e.g., some radioconjugates targeting the bombesin [10] or somatostatin [11] receptors. In the case of CP04-based radiopharmaceuticals, Roosenburg et al. [12] have found apparent IC_50_ variations of rather moderate character in compounds labeled with [^nat^In], [^nat^Ga], and [^nat^Cu] or non-labeled. Our previous study showed different chromatographic behaviors of Ga-CP04 complex compared to Lu-CP04 (HPLC-relative retention to CP04: 1.08 and 0.85, respectively). A different coordination of these metal ions was also confirmed by LC-MS study [13]. In the present contribution, we supplement these findings by showing that no significant difference in competition binding and cellular uptake and internalization exists between Ga^3+^ and Lu^3+^ chelated CP04. Furthermore, we report that the metal exchange has no influence on the peptide conformation. We also propose the structural basis for the CP04-CCK2R binding based on molecular modeling of the peptide-ligand interactions.

## Methods

### Chemicals

CP04 (DOTA-(DGlu)_6_-Ala-Tyr-Gly-Trp-Met-Asp-Phe-NH_2_) in GMP grade was purchased from piCHEM (Graz, Austria). The peptide was complexed with gallium and lutetium following the method described in our previous publication [13].

### Cell culture

The A431 human epidermoid carcinoma cell line stably transfected with the human CCK2R (A431-CCK2R(+)) and mock transfected with an empty vector alone (A431-CCK2R(−)) were kindly provided by Prof. Luigi Aloj [14].

The cells were cultured in Dulbecco’s modified Eagle’s medium (DMEM) supplemented with 10% of heat-inactivated fetal bovine serum and antibiotics (penicillin 100 U/mL and streptomycin 100 μg/mL) and cultivated at 37 °C in 5% CO_2_.

### Competition binding assay

The binding affinity (the apparent 50% inhibitory concentration (IC_50_)) of [^nat^Ga]Ga-CP04 and [^nat^Lu]Lu-CP04 complexes for CCK2 receptor was determined in A431-CCK2R(+) cell line using [^90^Y]Y-CP04 as a competitive radioligand according to the method used by Roosenburg et al. [12]. [^90^Y]Y-CP04 was obtained with a molar activity of 2.05 GBq/μmol calculated at the time of experiment and 98% radiochemical purity as determined by radio HPLC method. Human epidermoid carcinoma (A431-CCK2(+)) cells were subcultured overnight in 12-well plates in concentration of 0.5 mln per well. Before the experiments, the cells were washed with DMEM medium and then [^90^Y]Y-labeled CP04 (2.05 GBq/μmol) was added to the cells in amount of 0.24 pmol per well (ca. 80,000 cpm) and incubated for 1 h at 37 °C with increasing concentrations (10^−6^–10^−13^ M) of the Ga and Lu complexes of CP04 peptide. After the incubation, the medium was removed and the cells were washed with phosphate-buffered saline (PBS). Subsequently, the cells were lysed with 1 M NaOH and collected and the cell-associated radioactivity was measured using a well-type gamma counter (1470 Wizard, Wallac). The IC_50_ value (half maximal inhibitory concentration) was calculated based on competition nonlinear binding curve using GraphPad Prism software Version 7.03. The results are expressed as a mean ± SD of three experiments performed in triplicate.

### Cellular uptake and internalization

Cellular uptake/internalization of [^68^Ga]Ga- or [^177^Lu]Lu-labeled CP04 complexes in CCK2R-positive cells was carried out according to the method by Kaloudi et al. [15]. Approximately 100,000 cpm of [^68^Ga]Ga- or [^177^Lu]Lu-labeled CP04 (ca 1 ng of CP04/well) were added to the pre-incubated (24 h) cells (1 million cells in 2 mL of the medium) seeded in 6-well plates. The cells were incubated for 1 h at 37 °C. After incubation, the medium was removed and the cells were washed with cold 0.5% BSA-PBS. The binding to the cell surface receptors was determined by measurement of radioactivity of 1 mL of glycine buffer used for rinsing of cells. Then, 1 mL of 1 M NaOH was used for the lysis of the washed cells. Samples of the membrane-bound fractions and the internalized fractions (lysates) were measured for their radioactivity content using a well-type gamma counter (1470 Wizard, Wallac), and then, the percentage of membrane-bound and internalized fractions was calculated. The specific uptake to CCK2R receptors was calculated by subtracting the values obtained for A431-CCK2R(−) cells from the values obtained for A431-CCK2R(+) cells. At least four independent experiments were performed in triplicate.

### NMR measurements

For the purpose of NMR measurements, samples of the CP04 complexes were dissolved in 90% H_2_O/10% D_2_O (total volume of about 0.6 ml). The NMR spectra with WATERGATE solvent suppression were recorded at 400.13 MHz proton frequency and at 303 K on a Bruker DRX 400 spectrometer. The 1D ^1^H-NMR spectra were recorded according to standard procedures with a time domain of 32-k data points and a spectral width of 6410.27 Hz [16–21]. The free-induction decay was acquired for 2.556 s with a dwell time set to 78.0 μsec. The relaxation delay was set to 1 s. The sweep width of the 2D homonuclear TOCSY and ROESY spectra was 5592.84 Hz in the direct and indirect 1H-dimension, respectively. The free-induction decay was acquired for 183.1 msec with a dwell time set to 89.4 μsec. The relaxation delay was also set to 1 s. All spectra were zerofilled prior to Fourier transformation, and sine apodisation functions were applied in both dimensions using MestReNova [22].

### Homology modeling

The CCK2 receptor structure was obtained via homology modeling by using the SWISS-MODEL Homology Modeling server [23–25]. The template was the structure of δ-opioid receptor bound to naltrindole (PDB accession code: 4EJ4) which was selected based on sequence identity, similarity, and coverage as suggested by the server (no user intervention). All the default parameters were retained.

### Molecular docking

Several conformations of the unlabeled CP04 were generated and then docked to the CCK-2R homology model by using AutoDock Vina [26]. The binding box was selected to encompass the intrahelical cavity as well as extracellular loops and then extended to free volume around the receptor outlet; exhaustiveness was set to 20. The best scored binding poses were manually inspected for compatibility with mutagenesis data reported for CCK derivatives binding to CCK-2R [27].

## Results and discussion

### In vitro binding properties

With the purpose of checking the sensitivity of CCK-2R/CP04 interaction to the change of metal coupled to the peptide, [^177^Lu]Lu- and [^68^Ga]Ga-CP04 complexes were prepared and assayed for affinity to human CCK-2R by competition binding experiments in A431-CCK2R(+) cells. In both cases, the apparent affinity for hCCK-2R was in the low nanomolar range and was found comparable for [^nat^Ga]Ga-CP04 and [^nat^Lu]Lu-CP04 (IC_50_= 1.15 ± 0.39 nM, *n* = 3, and 1.02 ± 0.28 nM, *n* = 3, respectively).

Both complexes efficiently internalized in A431-CCK-2R(+) cells after 1-h incubation at 37 °C, showing a minor, 4% portion of radioactivity bound on the cell membrane, as expected for receptor agonists, with high internalization (over 70%) (Table 1). There was no statistically significant difference between the [^177^Lu]Lu- and [^68^Ga]Ga-CP04 complexes with regard to cellular uptake (according to the Student *t* test).

**Table 1:** Cellular uptake and internalization of radiolabeled CP04 complexes

	[^177^Lu]Lu-CP04	[^68^Ga]Ga-CP04
*n*	6	4
Membrane-bound fraction [%]	4.10 ± 0.91	3.63 ± 0.78
Internalization [%]	74.82 ± 7.74	73.45 ± 10.22

These observations prompted us to investigate their structural basis by NMR and molecular modeling.

### NMR studies

**Fig. 2:**
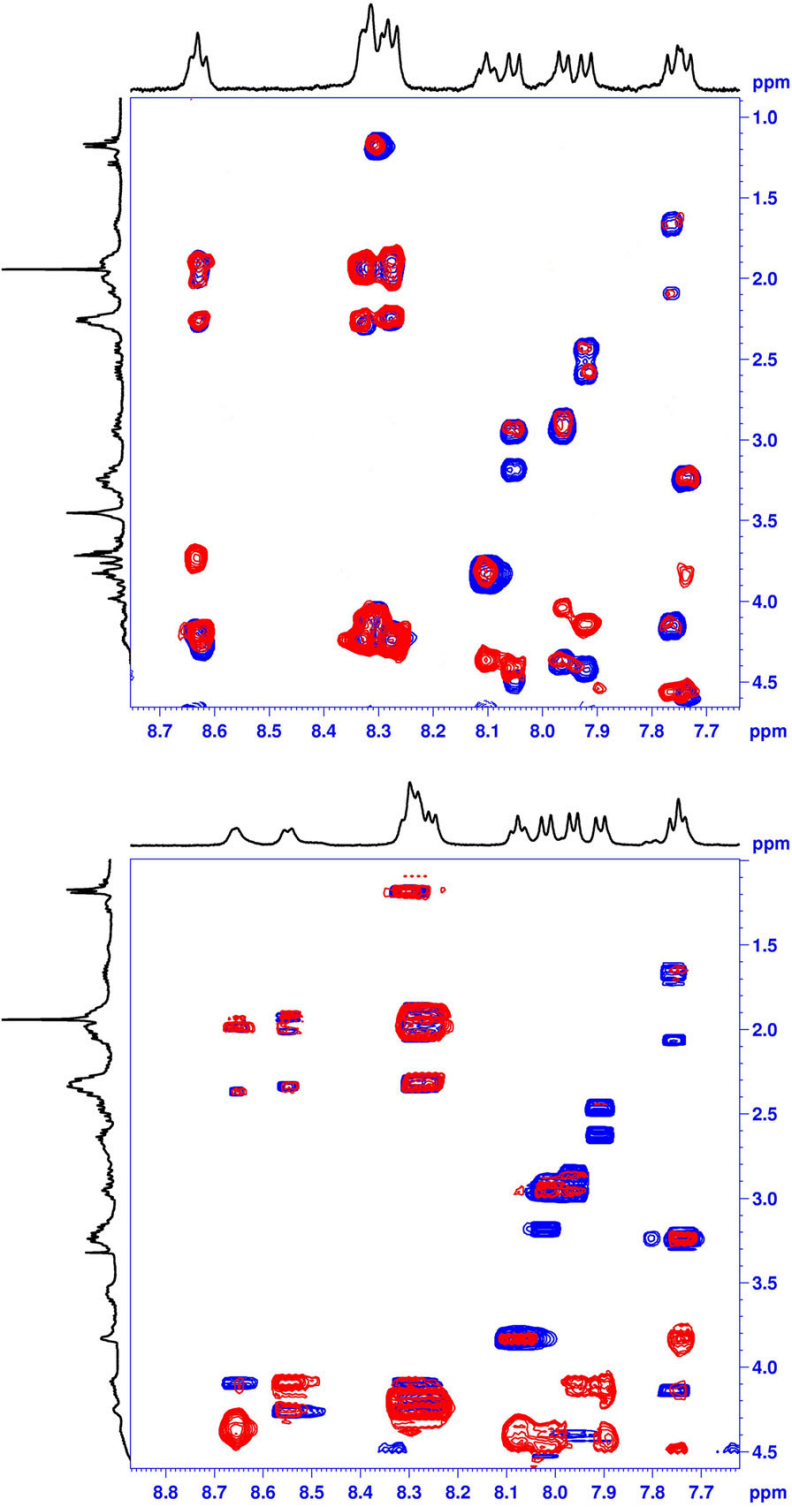
The overlay of the ^1^H-^1^H TOCSY (blue) and ROESY (red) spectra of Ga^3+^-CP04 complex (upper spectra) and Lu^3+^-CP04 complex (lower spectra). The red spots with no blue ones below indicate ROESY correlations between distinct amino acids

The complexes were subject to NMR TOCSY and ROESY experiments so to see whether the type of metal influences the structure of the compound. The signal dispersion in the 1D ^1^H NMR spectra (Fig. 2) suggests a rather dynamic conformational ensemble of molecules in solution. The respective ^1^H NMR signals were assigned on the basis of their characteristic chemical shifts, as well as TOCSY and ROESY correlations (Table 2). Most of the chemical shifts originating from amino acids in both complexes are almost exactly the same, except for those very close to the site of complexation, i.e., originating from the two last glutamic acid moieties. As a result, it can be concluded that no conformational difference between the two complexes is observed. The differences in the chemical shifts of the D-Glu^1–2^ can be ascribed to different coordination structures. In the crystal structure of [Ga-DOTA-D-Phe-NH_2_], the ion is coordinated by only two carboxylate groups, with the remaining carboxylate arm pendant [28]. Contrarily, no free carboxylate is found in DOTA (or DOTA derivatives) crystal structures with lanthanides (like Lu(III)) [28, 29]. The presence or lack of the free carboxylate group in the chelator moiety, as well as different geometries of the macrocycle bringing about local perturbation in a structure, is reflected by different chemical shifts corresponding to the amino acids neighboring the DOTA moiety.

**Table 2:** ^1^H chemical shifts (in ppm) of the Ga^3+^-CP04 and Lu^3+^-CP04 complexes

Complex	Amino acid	NH	Hα	Other H
Ga^3+^-CP04	D-Glu^1^	8.64	4.21	1.90, 2.27
Lu^3+^-CP04		8.66	4.09	1.99, 2.36
Ga^3+^-CP04	D-Glu^2^	8.62	4.26	1.90, 2.27
Lu^3+^-CP04		8.55	4.25	1.91, 2.02, 2.34
Ga^3+^-CP04	D-Glu^3^	8.28	4.24	1.88, 1.96, 2.25
Lu^3+^-CP04		8.29	4.26	1.95, 2.00, 2.31
Ga^3+^-CP04	D-Glu^4^	8.28	4.24	1.88, 1.96, 2.25
Lu^3+^-CP04		8.29	4.26	1.95, 2.00, 2.31
Ga^3+^-CP04	D-Glu^5^	8.33	4.22	1.93, 2.29
Lu^3+^-CP04		8.25	4.18	1.95, 2.30
Ga^3+^-CP04	D-Glu^6^	8.33	4.22	1.93, 2.29
Lu^3+^-CP04		8.25	4.18	1.95, 2.30
Ga^3+^-CP04	Ala^7^	8.30	4.11	1.18
Lu^3+^-CP04		8.30	4.09	1.18
Ga^3+^-CP04	Tyr^8^	7.96	4.37	2.89, 2.95, 6.79, 7.07
Lu^3+^-CP04		7.96	4.39	2.85, 2.94, 6.80, 7.08
Ga^3+^-CP04	Gly^9^	8.10	3.82	
Lu^3+^-CP04		8.08	3.83	
Ga^3+^-CP04	Trp^10^	7.74	4.59	3.24, 7.17, 7.20, 7.53, 10.10 (NH)
Lu^3+^-CP04		7.74	4.63	3.24, 7.10, 7.21, 7.54, 10.09 (NH)
Ga^3+^-CP04	Met^11^	7.76	4.15	2.09, 1.66
Lu^3+^-CP04		7.76	4.14	2.06, 1.67
Ga^3+^-CP04	Asp^12^	7.92	4.42	2.43, 2.58
Lu^3+^-CP04		7.91	4.42	2.46, 2.62
Ga^3+^-CP04	Phe^13^	8.05	4.49	2.95, 3.19, 7.15, 7.43
Lu^3+^-CP04		8.02	4.51	2.94, 3.17, 7.11, 7.44

A close inspection of the ROESY correlations indicates that only intraresidual and (a few) sequential cross correlations between amino acids could be detected (please refer to the overlay of ROESY and TOCSY spectra, Fig. 2). Therefore, no secondary structure can be identified, which further confirms that both complexes form a dynamic ensemble of conformations rather than an organized rigid structure.

### CP04 binding mode from molecular docking

**Fig. 3:**
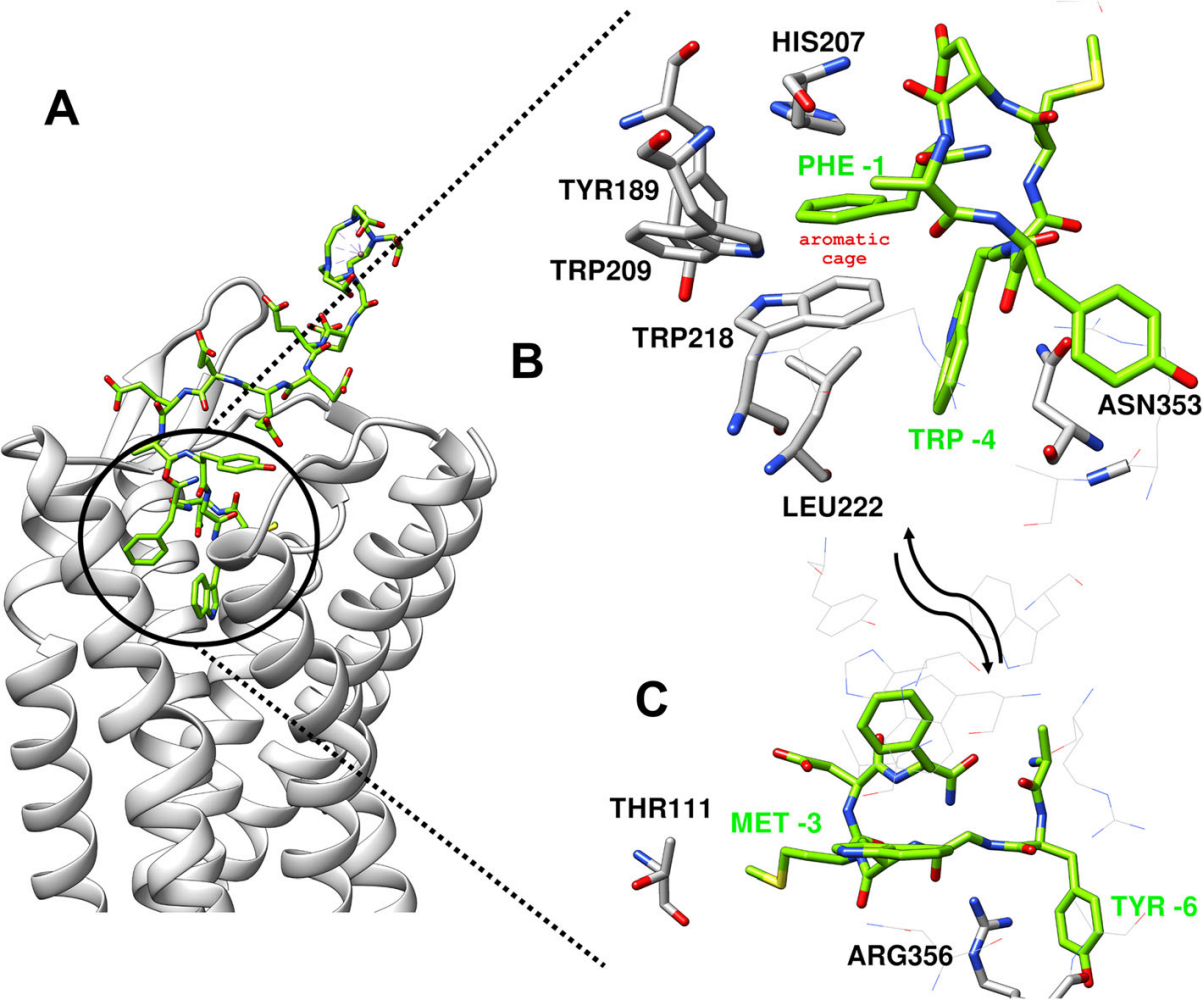
CP04 (green) docked to the homology model of the CCK2 receptor (gray): (**a**) general view and (**b**, **c**) focus on the interactions of the four C-terminal residues with the receptor presented from two perspectives

In order to elucidate the molecular basis of the CP04/CCK-2R interactions, the conjugate was docked to the receptor homology model (no CCK-2R crystal structure has been reported so far). The molecular docking predicts that CP04 enters the CCK2R binding pocket with the C-terminus directed towards the intracellular side (Fig. 3). Numerous contacts between the peptide and the receptor are created. Discussing these (as well as in further parts of the manuscript), for the sake of convenience, the CP04 amino acid residues are counted back starting from the C-terminus and the number denoted with the minus sign in the superscript. The aromatic side chain of the C-terminal Phe^−1^ is located in the “aromatic box” formed by Trp218^5.39^, His207^ECL2^, Tyr189^4.61^, and Trp209^ECL2^. The phenyl ring lies parallel to the rings of Trp218^5.39^ and His207^ECL2^, while perpendicular to the ring of Tyr189^4.61^. The charged side chain of the penultimate Asp^−2^ points to His207^ECL2^. The positioning of Met^−3^ side chain is less tight. The side chain is oriented towards Thr111^2.60^, but as there is a lot of free volume around it, it is probable that multiple orientations co-exist. In the case of Trp^−4^, the aromatic indole forms hydrophobic interactions with Trp218^5.39^, Leu222^5.43^ and Asn353^6.55^. A cation-π interaction is formed between Arg356^6.58^ and the aromatic ring of Tyr^−6^. Additional stabilization of the bound conformation may come here from a hydrogen bond between the Tyr^−6^ phenol function and the carbonyl of Ala357^6.59^.

The further (counting from the C-terminus) amino acid residues (including the D-Glu’s of the “spacer”) and DOTA are located at the binding site outlet where the receptor helices are located quite far from each other. Large free volume in this vicinity, solvent exposition, and a simultaneous lack of strong binding partners for flexible residues of the spacer arm make it likely that this part of CP04 assumes many conformations without sharp energetic minima.

### The proposed binding mode fits the SAR of CCK/gastrin derivatives and MG-based radiopharmaceuticals

**Fig. 4:**
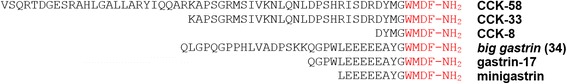
Sequences of the endogenous CCK-2R ligands. C-terminal WMDF-NH_2_ sequence is pharmacophoric for this receptor

Endogenous CCK-2R ligands include several cholecystokinin (CCK) and gastrin peptides of varying length (Fig. 4). In the past, their sequences were starting points for development of many peptide CCK-2R agonists. As a consequence, rich structure-activity relationship (SAR) data is available.

The presented binding model of CP04 to CCK-2R fits the structure-activity relationships of the peptide agonists of this receptor (on the assumption that CP04 binding mode overlaps with that of other derivatives). Especially, the positioning of the pharmacophoric C-terminal WMDF-NH_2_ seems to correspond well to the observations gathered in SAR studies (Table 3).

**Table 3:** SAR data from CCK/gastrin derivatives and their correspondence to our model

Residue	SAR data from CCK/gastrin derivatives	Correspondence to the in silico prediction	SAR ref.
C-terminal tetrapeptide	1. C-terminal sequence in cholecystokinins and gastrins is a condition necessary and sufficient for high affinity towards the receptor 2. Even a fragment as small as a tetrapeptide H-Trp-Met-Asp-Phe-NH_2_ (CCK-4) exhibits relatively good binding	The C-terminus entering the binding cavity in the helical bundle (as found in our model) fits this observation	[27, 48]
Phe^−1^	Exchange for a wide array of aromatic and aliphatic side chains is well-tolerated; it can even bring about a significant increase of affinity	The side chain in the “aromatic box” forms non-specific dispersive interactions, relatively bulky aliphatic or aromatic groups could be adapted	[49, 50]
Asp^−2^	Direct interaction of Asp with His207^ECL2^ been proposed earlier based on experimental results	Direct agreement—the charged side-chain points to His207^ECL2^	[51]
Met^−3^	1. The receptor affinity is indifferent to exchange for an equally long hydrophobic side chain of norleucine 2. Less favorable but still tolerable seems the introduction of phenylalanine, phenylglycine, or cyclohexylglycine 3. Short Ala side chain in this position brings about a notable decrease in affinity 4. The drop is dramatic upon introduction of charged ornithine or glutamic acid side chains (similar to Met regarding the length)	The side chain lies in a rather non-polar area with some free space around it allowing for example accommodation of a phenyl ring but not of the charged side chains	[50, 52]
Trp^−4^	1. A change in stereochemistry results in a binding decrease 2. This position is quite tolerant to some (but not all) substitutions for other l-aminoacids with bicylic aromatic side chains 3. Monocyclic aromatics like phenylalanine derivatives cause a decrease in affinity	1. Other side-chain directionality of D-Trp^−4^ analogues should require adaptation of a completely different binding mode 2. Smaller cycles should give less tight matching and some lowering of the affinity	[50, 53] [53, 54]

The herein reported binding mode can be also used to rationalize much of the binding data reported so far for minigastrin-based vectors and radiopharmaceuticals. In the light of our model, it is well understood why the chelating moiety has to be attached to the N-terminus while the derivatives with the ligand coupled via the C-terminal amide or in the para position of the Phe^−1^ exhibit a significant decrease in affinity of even several orders of magnitude [30]. A voluminous moiety at the C-terminus cannot be fit in the binding pocket together with the Trp-Met-Asp-Phe-NH_2_ and requires a complete rearrangement of the binding mode.

Analogously to the CCK and MG derivatives prepared for general medicinal chemistry, the MG-based vectors are most sensitive to the changes in the final C-terminal tetrapeptide sequence and the similar exchanges are tolerated (e.g., Met^−3^ to norleucine [31] or methoxinine [32]). Variations in the spacer residues between the chelator and the Trp-Met-Asp-Phe-NH_2_ influence the affinity only to a minor extent [4]. In the past, the derivatives with spacers of different lengths were reported (1–6 residues [31, 33, 34]) and with different residues (His [31], Gln, Ser, PEG [34]) instead of the hexa-Glu sequence. All these variations had only little impact on binding. The same applies to the stereochemistry of the spacer as the hexa-L-Glu derivative has similar affinity as the hexa-D-Glu analogue [7]. The derivatives with cyclized (rigidified) elements in the spacer do not have a much improved affinity [35]. Furthermore, little impact on affinity is seen also with different chelating agents at the N-terminus (DOTA, DOTAGA, DTPA, HYNIC) [30, 33, 35–37]. The presence and the character of metal give none but small changes in the binding strength. Some minigastrin analogues complexed with ^nat^In have been reported to have IC_50_ identical (within error) to the one of metal-free conjugates [7, 34]. Roosenburg and colleagues prepared CP04 analogues with three different chelators unmetaled or in complex with three different metals (12 variants in total), and the IC_50_ values varied between 0.8 and 1.5 nM [12]. In the contribution herein presented we see no significant difference in binding of compounds with Ga and Lu even though Ga^3+^-DOTA and Lu^3+^-DOTA have different coordination structures (one free carboxylate arm in the chelate with Ga^3+^ [28, 29]). The affinity of shorter minigastrin analogue with AAZTA as a chelator (AAZTA-MG) also did not exhibit sensitivity with regard to the incorporated metal [38].

The relative insensitivity of the binding affinity to the changes in the spacer, chelating moiety, and the presence/absence of metal as well as its exact type is again understandable within our binding model. The N-terminal part of CP04 (and by analogy: N-terminal part of the discussed radiopharmaceuticals) is predicted to be located at the receptor binding site outlet. Due to a large free volume in this vicinity, lack of strong interaction partners, as well as the solvent exposure of the site, accompanied by significant flexibility of the peptide chain, it is not unreasonable to suppose that this substructure may take several energetically similar conformations. With a few poses in equilibrium and no tight arrangement of the N-terminus, different modifications (spacer length, exchange of amino acids in the spacer, cyclization, and various chelators and metals) do not disturb the binding to a significant degree. For example, if any electrostatic mismatch lowering affinity might occur upon a particular structural change in the conjugate, the molecule should be easily able to accommodate a different binding pose avoiding unfavorable contacts. And contrarily, a possible additional drug-receptor contact in this vicinity should not improve the binding a lot since in the solvent-exposed environment, the strength of a single intermolecular electrostatic interaction is reduced. The experimentally observed small variations are too subtle to be explained or predicted by the used modeling methods. An attempt of such prediction would require also incorporation of the amino terminal domain of the receptor (absent in this model and in many crystallographic structures of GPCRs; CCK-2R N-terminus shows no significant homology to any protein found in the PDB database [39]).

### The proposed binding mode in the light of earlier receptor studies

Extensive experimental studies had been performed on CCK2 receptor in order to determine the structural basis of the interactions with ligands. Unfortunately, not all of them coincide with our predictions. Recently, based on photoaffinity labeling studies and hints from mutagenesis, it has been proposed that Phe120^ECL1^ interacts with Phe^−1^ of a CCK-like probe compound [40]. Contrarily, according to our binding pattern, this residue does not have any direct interaction with CP04. In the past, photoaffinity labeling studies suggested also Phe122^ECL1^ and Thr119^ECL1^ to be involved in interactions with CCK [41]; however, in our model, no contact with CP04 is predicted. On the other hand, in agreement with the results presented here, fluorescence spectroscopy studies supported the entrance of the CCK C-terminus into the intramembranous helical bundle [42].

Regarding the site-directed mutagenesis, some of the reported data do not seem to fit our model. The mutation of residues such as Arg57^1.35^, Tyr61^1.39^, Phe120^ECL1^, Thr193^4.65^, Trp346^6.48^, and Val349^6.51^ has been shown to influence more or less the binding affinity of cholecystokinin peptide [27]. In the binding mode presented herein, these residues are rather distant from any of the CP04 residues. Still, several other results from mutagenesis are easily reconcilable with our model (Table 4).

**Table 4:** CCK-2R mutagenesis data and their correspondence to our model (ref. [27] and references therein)

Receptor residue	Mutation	Impact on CCK/gastrin affinity for CCK-2R	Our in silico model
Thr111^2.61^	T111A	Decrease	The residue in the vicinity of Met^−3^, exchange to Ala gives more void and less tight matching
Met134^3.32^	M134A	Slight increase	Met-3 side chain is predicted to be positioned so that the branched methyl unit of Leu would create a steric clash, however relatively ease to relieve
	M134L	Slight decrease	
Tyr189^4.61^	Y189A	Drastic drop of CCK affinity	The residue involved in the “aromatic cage,” it interacts with Phe^−1^
His207^ECL2^	H207A	Significant decrease in CCK affinity	Earlier reports suggested the interaction between His207^ECL2^ and Asp^−2^ of CCK, such a contact is present in our model
Asn353^6.55^	N353L	More hydrophobic Leu improves CCK and gastrin affinity	Participates in hydrophobic contacts with Trp^−4^
	N353A	Shorter Ala decreases the affinity	
Arg356^6.58^	R356D	Large drop in CCK affinity	The residue involved in cation-π interactions, exchange for negatively charged side chain (R->D) should disrupt this interaction, shorter positively charged (R->K/H) or neutral residues (R->A) should bring about some decrease in affinity
	R356A	Decrease less dramatic compared to R356D	
	R356K/H	Decrease less dramatic compared to R356D	

Here let us note that all comparisons between the experimental data and our model are burdened with several issues. First of all, most of the experimental studies were performed for cholecystokinin or variants thereof. While the C-terminal residues are identical in those and in CP04, the remaining parts of the molecules may influence the binding mode of the common sequences. Secondly, the loss of affinity upon a mutation could mean that a particular residue is involved in interactions with the ligand in the final binding pose, but it cannot be excluded that such a residue only takes part in the process of ligand entrance or induced-fit. The same applies to the results coming from the photoaffinity labeling or fluorescence spectroscopy.

In our binding mode, the positioning of the C-terminal tetrapeptide sequence differs considerably from the one proposed for cholecystokinin in earlier studies [43, 44]. These differences seem inevitable as the earlier homology models had been built on rhodopsin or β-adrenergic receptor as templates. Our model uses the inactive crystal of delta opioid receptor. This structure and those ones differ as to the relative positions of the helices and the geometries of the loops, and consequently, the binding sites do differ too. However, our prediction agrees with those earlier propositions in that the terminal tetrapeptide sequence enters the cavity formed between the helices. For the sake of completeness, let us mention here that other homology models have been reported too [45, 46], but as they were used for modeling of small molecular binders, no comparisons can be made here.

## Conclusions

Although usually the modeling and docking studies are employed in the design of new molecules, in this work, we referred to them from the radiopharmacy routine perspective in order to better understand the tracer’s biological behavior. The structure of DOTA complex is not the same when either Ga^3+^ or Lu^3+^ is incorporated; however, our competition binding assay as well as cellular uptake and internalization studies demonstrated that there is no significant difference in affinity for CCK-2R between the Ga- and Lu-bearing CP04 complexes. NMR spectroscopy shows that the metal influences only the conformation of the direct neighborhood of the chelating site. We also propose a binding mode for CP04 and CCK-2R based on homology modeling of the receptor and molecular docking. In this model, the conjugate dips down between the helices with the C-terminal end, while the N-terminal part including DOTA and the metal is at the receptor outlet. The residues of the carboxy terminus form many well-defined interactions with the receptor binding site. Much of these seem consistent with experimental SAR data for cholecystokinin and derivatives. Our in silico model allows also the rationalization of the so far reported binding data for minigastrin-based radiopharmaceuticals. It also partially fits the experimental receptor studies (mainly mutagenesis).
